# Chromosomal alteration patterns in PitNETs: massive losses in aggressive tumors

**DOI:** 10.1530/ERC-24-0070

**Published:** 2024-12-19

**Authors:** Maaia Margo Jentus, Leontine Bakker, Marco Verstegen, Iris Pelsma, Tom van Wezel, Dina Ruano, Ellen Kapiteijn, Stijn Crobach, Nienke Biermasz, Hans Morreau

**Affiliations:** 1 Department of Pathology, Leiden University Medical Center, Leiden, The Netherlands; 2 Department of Medicine, Division of Endocrinology, Leiden University Medical Center, Leiden, The Netherlands; ^3^ Center for Endocrine Tumors Leiden (CETL), Pituitary Center, Leiden University Medical Center, Leiden, The Netherlands; 4 Department of Neurosurgery, Center for Endocrine Tumors Leiden (CETL), Leiden University Medical Center, Leiden, The Netherlands; 5 Department of Medical Oncology, Leiden University Medical Center, Leiden, The Netherlands

**Keywords:** pituitary tumor, PitNET, PitNETs, prolactinoma, molecular diagnostics, copy number alterations, chromosome alterations, loss of heterozygosity, CNV, LOH, cnLOH, SNP analysis, endoreduplication, genome doubling, genome haploidization, near-haploidization, near-homozygous genome, near-haploid genome, endocrine pathology

## Abstract

The molecular biology of pituitary neuroendocrine tumors (PitNETs) revealed few recurrent mutations and extensive chromosomal alterations, with the latter being the driving force in a subset of these lesions. Addressing the need for an easily applicable diagnostic tool, we conducted a retrospective study of 61 PitNETs operated at a tertiary care center. All cases were subtyped according to the 2022 WHO Classification of Endocrine Tumors. A genome-wide next-generation sequencing panel targeting 1500 single nucleotide polymorphisms (SNPs) was used to classify chromosomal imbalances, loss of heterozygosity, and copy number variations in DNA from formalin-fixed paraffin-embedded tissues. We identified four distinct chromosomal patterns, with varying distribution among different tumor lineages. Forty-two of 61 (69%) PitNETs showed chromosomal alterations. Gonadotroph PitNETs showed mostly quiet genomes. The majority of lactotroph PitNETs (19/20, 95%) were altered, exhibiting a gained genome and a remarkably low recurrence rate. Nine of ten (90%) corticotroph PitNETs harbored chromosomal alterations, of which two aggressive corticotroph tumors and one metastatic corticotroph PitNET showed massive chromosomal losses, leading to near-haploid/near-homozygous genomes. The comparison of the molecular profile of primary and recurrent PitNETs of five patients showed no significant accumulation of alterations over time. A simple genome-wide 1500-SNP test can be used in the identification of outspoken aggressive subsets of PitNETs by the occurrence of a near-haploid/near-homozygous genome. Furthermore, the presence of neoplastic tissue in the resected material can be potentially confirmed for non-gonadotroph PitNETs under suboptimal histological assessment conditions.

## Introduction

Pituitary neuroendocrine tumors (PitNETs), previously named pituitary adenomas, frequently require lifelong medication and/or recurrent surgeries with associated risks of side effects and complications. Although being histologically mostly ‘low grade’, some cause invasive growth, making radical resection impossible and thereby necessitating local radiotherapy in case of relevant growth despite surgery (Raverot *et al.* 2018). Metastases are uncommon, but metastatic or aggressive potential is yet unpredictable. The novel WHO terminology excludes the term ‘carcinoma’ for aggressive or metastatic PitNETs, voicing that this term should be reserved for poorly differentiated neuroendocrine carcinoma. Current guidelines define aggressive pituitary tumors on the basis of clinical parameters, with limited evidence guiding diagnostic and therapeutic procedures (Raverot *et al.* 2018). No widely accepted molecular markers exist that significantly change clinical management, including surveillance strategy and early recognition of particularly aggressive cases. Obtaining family history and excluding an underlying genetic syndrome are important (Villa *et al.* 2019). Histological subtype, age, and radiological grade of the tumor predict the risk of recurrence (Trouillas *et al.* 2013, Gomez-Hernandez *et al.* 2015, Asa & Ezzat 2022, Villa *et al.* 2023). Further observations suggest that men with lactotroph tumors tend to have a worse prognosis (Delgrange *et al.* 2015).

Histological grading systems for PitNETs lack standards, unlike those for other neuroendocrine tumors, in which proliferative rate defines grade (Trouillas *et al.* 2020). Metastatic and non-metastatic PitNETs often are histologically similar. A high Ki67 proliferation index is no reliable marker for metastatic potential, even though multiple studies found associations between Ki67 and invasive growth (Mete *et al.* 2012). Crooke cell tumors, acidophilic stem cell tumors, immature PIT1-lineage tumors, and sparsely granulated somatotroph tumors show adverse behavior (Asa *et al.* 2021, Villa *et al.* 2023). There is no consensus on whether silent corticotroph tumors truly represent a high-risk type (Gomez-Hernandez *et al.* 2015, Fountas *et al.* 2018).

Sporadic PitNETs show few recurrent DNA mutations, with increased frequencies in genes such as *USP8* in corticotroph tumors, *GNAS* in somatotroph tumors, and *TP53*, *DAXX*, and *PTTG* in aggressive cases (De Sousa & McCormack 2022, Manojlovic-Gacic *et al.* 2018, Heaphy *et al.* 2020, Asa & Ezzat 2022). The relative lack of recurrent mutations prompted researchers to use genome-wide analysis to study PitNET biology (Simpson *et al.* 2003, Välimäki *et al.* 2015, Bi *et al.* 2017, Sapkota *et al.* 2017, De Sousa *et al.* 2019, Neou *et al.* 2020, Trouillas *et al.* 2020, Cui *et al.* 2021). PitNETs harbor extensive chromosomal alterations across the whole genome that differ in rate. Studies separated PitNETs into genomic quiet and disrupted groups, with variable subtyping based on chromosomal imbalance with copy number gain, imbalance with copy number loss, loss of heterozygosity (LOH), and the occurrence of ‘tetraploid’ chromosomes. A comparison of historical and current studies is challenging due to continuous changes in nomenclature and the variable use of immunohistochemistry for tumor subtyping, as well as the applied molecular methods (Supplementary Fig. 2A and B, see section on supplementary materials given at the end of this article).

When comparative genomic hybridization (CGH)-based gene copy number counting, fluorescent *in situ* hybridization, and, sometimes, conventional cytogenetics are used as an analysis tool, copy-neutral LOH will be missed and genome-wide haploidization is not recognized in CGH. In other words, if the normal AB genotype is altered in lesions into AA or BB, that alteration will not be detected. The detection of copy-neutral LOH requires allelic genotyping, which can be assessed through single nucleotide polymorphism (SNP) analysis and sequencing at a reasonable depth (whole-exome and whole-genome sequencing). In oncocytic thyroid carcinoma (OTC), the occurrence of chromosomal near-haploidization with or without endoreduplication/genome doubling as a driving force in the tumorigenesis was only discovered after the use of SNP typing techniques that could visualize the tumor genotypes (Corver *et al.* 2014, Ganly *et al.* 2018, Gopal *et al.* 2018). In OTC, the chromosome 7 gain reported when using CGH was, in fact, retention (genotype AB) of both alleles of this chromosome with the loss of most other chromosomes (genotype A0 or 0B). Endoreduplication/genome doubling led to genotypes AABB of obligatory preserved chromosome 7 and AA/BB in most other chromosomes (Boot *et al.* 2016).

We have recently reported on a clinically applicable next-generation sequencing (NGS) analysis including a bioinformatics pipeline of 1500 SNPs across all autosomes and the X chromosome in DNA derived from a formalin-fixed paraffin-embedded (FFPE) material. With this method, chromosomal imbalance/LOH and copy number variation (CNV) patterns were reliably analyzed in oncocytic thyroid (OT) neoplasms (de Koster *et al.* 2023). In the current study, we used the same approach in a histologically subtyped PitNET cohort and described comprehensive patterns of chromosomal imbalance/LOH and CNV to validate and systematize the heterogeneous data obtained from the literature and explore possible applications in routine diagnostics.

## Materials and methods

### Study design and case selection

For the current retrospective study, pseudo-anonymized pathology records were reviewed of 61 randomly selected PitNET cases and analyzed with genome-wide SNP analysis at our tertiary care center from 2012 to 2024. The analysis was performed on the primary tumor and/or recurrent tumors. All histopathological diagnoses were reviewed by endocrine pathologists (M Jentus and H Morreau) in accordance with the WHO Classification (5th edition, WHO 2022). An ethical study review approval was waived by the Medical Ethics Review Committee Leiden (G19.011). No informed consent was required.

### Data collection

Pathological data consisted of hematoxylin–eosin and immunohistochemical stained slides (Supplementary Material 1), data of the SNP analysis, and the subsequent molecular reports. Clinical data were collected by attending endocrinologists (L Bakker and N Biermasz) as suggested by the European Pituitary Pathology Group (Villa *et al.* 2019) as follows: endocrine status; preoperative medication and/or radiation therapy; type of medication; recurrence; preoperative MRI with maximal tumor dimensions in mm (micro-/macro-/giant tumor); and invasion status in cavernous sinus, sphenoidal sinus, and/or bone.

### Molecular analysis

For molecular testing, total nucleic acid was isolated from FFPE tissue sections after microdissection of serial hematoxylin-stained sections and selection of tumor tissue on the basis of hematoxylin and eosin-stained diagnostic slides, including immunohistochemical stained slides in difficult cases. We performed genome-wide imbalance/LOH/CNV analysis by sequencing 1500 SNPs across all autosomes and the X chromosome using targeted NGS, and data were analyzed as previously described (de Koster *et al.* 2023). When tumor cell percentage is sufficiently high (mostly the case in PitNETs), imbalances and LOH are identified from the SNP frequency patterns. Imbalances are then characterized by smaller amplitude changes when compared to LOH. Copy number detection by CNV analysis helps explain the mechanism behind the observed imbalances/LOH, being either chromosomal gains or losses. Subsequently, genotypes were extrapolated. The patterns observed across the patient cohort were scored, and the alterations were counted. For part of the aggressive cases, additional mutational analysis with diverse panels was performed (Supplementary Material 1). In one aggressive case, whole-genome sequencing (WGS) analysis was performed using a BioWDL reimplementation v3.2 (available at https://github.com/biowdl/WGSinCancerDiagnostics) of the Hartwig Medical Foundation’s WGS pipeline 5 (accessible at https://github.com/hartwigmedical/pipeline5).

### Statistical methods

Due to the limited number of the patients included in the study, there was insufficient statistical power to conduct robust inferential statistical analyses. The study primarily relied on descriptive statistical methods to analyze the data. The variable ‘total alterations’ was determined by calculating the sum of affected chromosome arms for every patient (41 arms maximum possible). All statistical analyses were performed using Prism 9.3.1 (GraphPad Software, USA). Where applicable, quantitative parameters are presented with minimum, maximum, median, and mean along with standard deviation (SD). Normality was assessed with Kolmogorov–Smirnov or Shapiro–Wilk’s test. Unpaired *t*-tests were used for comparison of two groups in normally distributed variables, the Mann–Whitney U test (Wilcoxon’s rank-sum test) was used for skewed data, and one-way ANOVA was applied for comparison of multiple groups. The chi-square test was utilized for contingency analysis. A significance level of *P* < 0.05 was considered statistically significant.

## Results

### Characteristics of the study cohort

**Figure 1:**
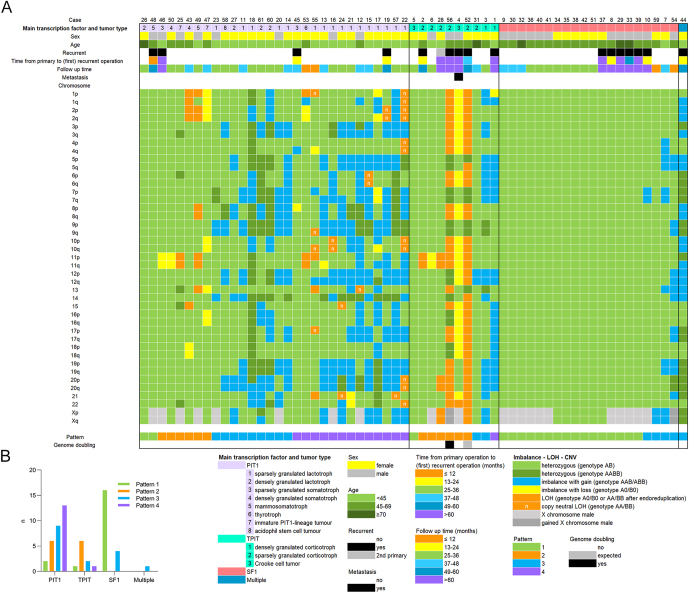
Overview of the cohort with selected clinical characteristics of the patients, PitNET subtypes, imbalance–LOH–CNV alterations noted per chromosomal arm, and corresponding patterns. (A) Cases are sorted by lineage and further by pattern, from the least number of disrupted chromosomes to those most affected. The chromosome arms with the highest frequency of alterations in the cohort were 12q and 12p, primarily exhibiting imbalances due to chromosomal copy number gain. Following chromosome 12, the frequently altered chromosomes in descending order of frequency are 20, 19, X, 3, 5, 7, and 8, showing imbalances due to copy number gain, while chromosome 11 predominantly exhibited LOH or imbalance due to copy number loss. Chromosome 1, frequently mentioned in the literature, displayed alterations in only 14 of the 61 PitNETs. (B) A simplified overview of the distribution of the observed chromosomal alteration patterns in different lineages of PitNETs.

Sixty-one cases were included in this study (Fig. 1). The cohort consisted of 23 male (37.7%) and 38 female patients (62.3%). The mean age of the cohort was 46 years (median 47 years, range 19–83). The cohort included 45 primary tumors and 16 recurrent tumors. One of the recurrent tumors was metastatic (patient 4). The tumors of the PIT1 lineage represented the largest group (30 patients, 49.2%), followed by 20 SF1-lineage PitNETs (32.8%), 10 tumors of TPIT (16.4%), and one multilineage PitNET (1.6%); PIT1 and SF1 positive with extensive immunohistochemical expression of growth hormone and sparsely expressed prolactin (Asa *et al.* 2023). One of the patients had a sparsely granulated lactotroph PitNET first and presented with a primary silent corticotroph tumor 7 years later (patient 28). In the category of PIT1-positive cases (*n* = 30), the majority were lactotroph PitNETs (*n* = 20, 65%). The somatotroph subgroup comprised four cases (13.3%). Mammosomatotroph and immature PIT1-lineage tumor subgroups each contained two patients (each 6.7%). Finally, only one thyrotroph and one acidophil stem cell tumor case were included. Of the 20 lactotroph tumors, 16 were sparsely granulated and 4 were densely granulated.

### Imbalance–LOH–CNV analysis: identification of distinct patterns

**Figure 2:**
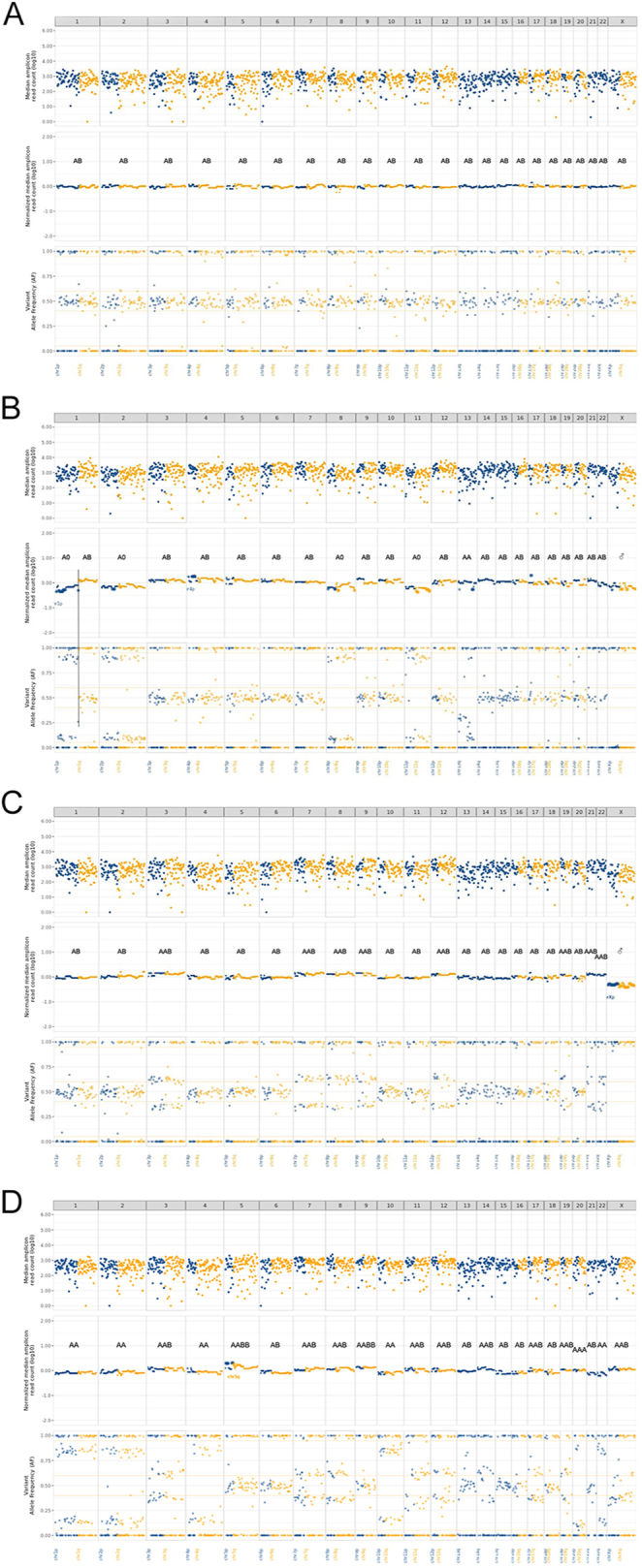
Examples of Patterns 1–4 as observed in the PitNET cohort (A–D). Scoring of informative SNPs resulted in the patterns of imbalance or loss of heterozygosity (LOH) combined with copy number variation (CNV) analysis across the entire study cohort. The upper section of such analysis shows the coverage of all 1500 SNPs used, the middle section represents the CN profiles, and the lower section depicts the allelic frequency for each SNP. Chromosome-wide genotypes were subsequently extrapolated and indicated as AB; A0 or 0B; and AA, BB, and AABB, the latter three occurring after genome doubling or endoreduplication. In some cases, locus-restricted copy-neutral LOH can be observed (also carrying genotype AA or BB). Chromosomal gains with imbalances in the SNP typing exhibit genotype AAB or ABB. (A) Example of Pattern 1: patient 51, a female patient with a primary silent gonadotroph PitNET, showing no imbalance or LOH or CNV alterations on any of the chromosomes, with heterozygous genotype of AB extrapolated. (B) Example of Pattern 2: patient 49, a male patient with a primary mammosomatotroph PitNET, exhibiting LOH of chromosomes 1p, 2, 8, 11, and 13 due to copy number loss, with an extrapolated genotype A0 or 0B. Chromosome 13 shows a difficult-to-interpret heterogeneous pattern with some imbalances with loss-like deviation and an extrapolated genotype of AA. There is only one chromosome X (male), genotypes A0 and 0B. (C) Example of Pattern 3: patient 20, a male patient with a primary sparsely granulated lactotroph PitNET, displaying extensive imbalances with copy number gain on chromosomes 3, 7, 8, 9, 12, 19, 21, and 22, with extrapolated genotypes AAB and ABB. There is again only one chromosome X (male). (D) Example of Pattern 4: patient 22, a female patient with a primary sparsely granulated lactotroph PitNET, exhibiting a complex CNV pattern with copy-neutral LOH on chromosomes 1, 2, 4, 10, 20, and 22 and imbalances with copy number gains on chromosomes 3, 7, 8, 11, 12, 14, 17, 19, and X, with extrapolated genotypes AA or BB and AAB or ABB, respectively. The copy-neutral LOH was scored as such because, in the copy number scoring in the middle panel, the height of chromosome 1 with LOH is similar to the heterozygous chromosome 6 (genotype AA vs AB, respectively). There is also a heterozygous state with whole chromosome gain on chromosomes 5 and 9 showing genotype AABB.

Following imbalance/LOH and CNV analysis (1500-SNP panel), four distinct patterns were identified (Fig. 2, panels A, B, C, D):

- Pattern 1: no alterations (with genotype AB).
- Pattern 2: LOH or imbalances caused by chromosome loss. Imbalances (with genotype A0 or 0B) are scored when the alterations in the SNP profiles show a smaller amplitude in comparison with LOH, which is scored below 0.25 and above 0.75 markings on the plots. LOH shows genotype A0 or 0B or AA or BB with, in the latter, an extensive amplitude in the SNP analysis and predicted endoreduplication/genome doubling if multiple arms are affected and all heterozygous chromosomes show AABB genotype. Locus-restricted copy-neutral LOH shows genotype AA or BB.
- Pattern 3: imbalances caused by chromosomal copy number gain (with genotype AAB or ABB).
- Pattern 4: heterogeneous pattern with mixed chromosomal copy number gains and losses.

Seen in all, but especially across Patterns 3 and 4, there are chromosomes with genotype AABB, concluded on the basis of the relative CNV information in the analysis. If present in all heterozygous chromosomes, this might be an indication of endoreduplication/genome doubling.

### Different chromosomal patterns observed between PIT1, TPIT, and SF1 transcription-positive PitNETs

Imbalance–LOH–CNV alterations were found in 42 of 61 PitNETs (69%, Patterns 2–4), and the remaining 19 (31%) showed no alterations (Pattern 1), as shown in Figs 1 and 2. The imbalance–LOH–CNV patterns were compiled in relation to primary subtyping based on transcription factors. TPIT-lineage tumors harbored most alterations, with an average of 14.1 affected chromosome arms (out of 41 arms; SD 13.04, 95% CI 4.77–23.43), followed by PIT1-lineage tumors with an average of 11.6 (SD 7.69, 95% CI 8.73–14.47), and the least affected were tumors of SF1 lineage with a mean of 1.4 (SD 3.69, 95% CI −0.33 to 3.13) altered chromosome arms. In Fig. 1 (panel A), the frequencies and types of alterations across the different chromosomes are represented. After sorting by lineage and molecular pattern, highly disrupted cases stand out. Heterozygous copy number gains (genotype AABB) were mostly observed in the PIT1-lineage group in varying frequencies. Patient 18 stood out with 27 chromosomal arms showing AABB genotype.

#### PIT1

The PIT1-lineage group (*n* = 30) showed 2× Pattern 1, 6× Pattern 2, 9× Pattern 3, and 13× Pattern 4 (Supplementary Table 1). Pattern 4 featured lactotroph PitNETs (*n* = 11), one sparsely granulated somatotroph PitNET, and one thyrotroph PitNET. Eight lactotrophs exhibited Pattern 3, while Pattern 2 was not observed. Pattern 2 in PIT1-lineage cases mostly included somatotrophs (3 of 4) and one mammosomatotroph, both of which are immature PIT1-lineage tumors. Lactotrophs were the most disrupted across all chromosomes (average of 15.1 chromosomal arms, SD 6.81, 95% CI 11.91–18.29), while sparsely granulated tumors had more alterations (average of 16.81 chromosomal arms, SD 6.06, 95% CI 13.58–20.04) compared with densely granulated tumors (average of 8.25 chromosomal arms, SD 5.68, 95% CI −0.79 to 17.29). While all the sparsely granulated lactotrophs harbored alterations, among the four densely granulated lactotroph tumors, three tumors exhibited Pattern 3 and one exhibited Pattern 1 (patient 26).

#### TPIT

The TPIT-lineage group (*n* = 10) showed 1× Pattern 1, 6× Pattern 2, 2× Pattern 3, and 1× Pattern 4. Corticotrophs exhibited an average of 13.7 altered chromosomal arms. One 19-year-old female patient (patient 5) displayed Pattern 1 phenotype in a Crooke cell tumor driven by a known *USP8* (exon 14) c.2152T>C, p. (Ser718Pro) somatic mutation found in additional analysis. Another patient with a Crooke cell tumor (patient 4, highlighted in the section ‘Selected cases with advanced disease’ of the supplementary material) demonstrated extensive Pattern 2 alterations. Among six sparsely granulated corticotroph PitNETs, five exhibited Pattern 2 alterations with one exception (patient 31) showing Pattern 3. Two densely granulated tumors showed Pattern 2 (patient 3) and Pattern 4 (patient 1).

#### SF1

Among gonadotrophs, most tumors (16/20) exhibited Pattern 1, while the four other tumors displayed Pattern 3.

### Radiological invasion status

Thirty-four tumors showed signs of radiological invasiveness, whereas 23 tumors were noninvasive, and for 4 tumors, no data were available. We found no significant difference in the quantity of LOH or imbalances in the invasive and noninvasive groups. The quantity of total alterations was normally distributed only in the noninvasive group. The invasive group harbored significantly less alterations than the noninvasive group (*P* = 0.0058, two-tailed Mann–Whitney U test). The patterns were significantly distributed among the invasion status (*P* = 0.0005, chi-square), with Patterns 1 and 2 more likely to be invasive and Pattern 4 more likely to be noninvasive. In tumors with Pattern 3, both invasive (*n* = 7) and noninvasive (*n* = 8) tumors were comparably observed.

### Functional status

In total, 22 tumors were clinically silent and 39 were functional. The silent group consisted of four sparsely granulated corticotroph PitNETs and 18 gonadotroph PitNETs. There was significant difference in functional status in different patterns (*P* < 0.0001, chi-square). Silent tumors were mostly seen with Pattern 1 (*n* = 16) and never with Pattern 4, whereas only three functional tumors showed Pattern 1, but most commonly exhibited Pattern 3 (*n* = 14), followed by Pattern 4 (*n* = 13) and Pattern 2 (*n* = 9). Three silent tumors showed Pattern 2, and three tumors showed Pattern 3. All four silent corticotroph tumors were sparsely granulated and invasive, but only one was reoperated. For 19 lactotroph tumors, serum levels of prolactin at the time of diagnosis were available. There was no significant relationship of prolactin levels neither with total chromosomal alterations or patterns nor with quantitative alterations of every described type.

### Therapy modalities and treatment decision in lactotroph PitNETs

Out of the 20 patients with lactotroph tumors (all functional), 18 received dopamine agonist (DA) therapy before surgery. Two patients did not receive DA therapy: one preferred surgery as the primary treatment (patient 15) and the other presented with a macrotumor with apoplexy and cranial nerve palsy (patient 20). Most medicated patients with lactotroph PitNETs underwent surgery due to DA intolerance or side effects (13 out of 18), rather than aggressive behavior or DA resistance (1 out of 18 patients, patient 61, with a microtumor showed a biochemically good response, but no tumor shrinkage and incomplete symptom relief). Another 4 out of 18 patients treated with DA opted for surgery instead of continuing medication. None of the 20 patients with lactotroph PitNETs received radiotherapy or temozolomide before surgery. One patient (patient 26, Pattern 1) was treated with radiotherapy after surgery due to a grade Knosp 3b giant tumor, which did not require repeated surgery at the 32-month follow-up.

### Recurrent PitNETs

**Figure 3:**
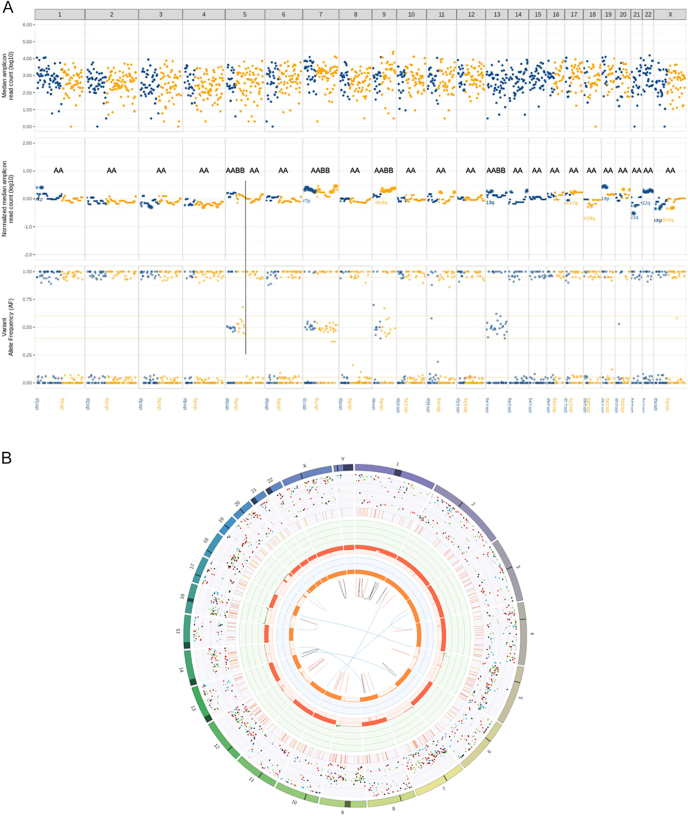
Near-haploid genome as seen with genome-wide 1500-SNP testing in the tumor of parent 52 (panel A) and with WGS in the tumor of patient 56 (panel B). (A) GW-LOH testing and imbalance–LOH–CNV analysis on tumor of patient 52: this testing shows a near-homozygous genome with multiple LOH (chromosomes 1, 2, 3, 4, part of 5q, 6, 8, 10, 11, 12, 14, 15, 16, 17, 18, 19, 20, 21, 22, and X), which is consistent with possible endoreduplication. These data fit with Pattern 2 of abnormalities. A previous recurrence of 4–5 years earlier shows an identical pattern of chromosomal alterations. (B) WGS result of case 56: LOH on chromosomes 1, 2, 3, 4, 6, 8, 10, 11, 13, 15, 17, 19, 20, 21, 22, and 23 can be observed. Molecular tumor cell percentage: 65%. The data point to Pattern 2 of chromosomal abnormalities. Endoreduplication occurred, leading to a near-homozygous genome (estimated ploidy 1.3).

The need of repeated operations was considered indicative of pathological/surgical recurrence, whereas the existence of persistent disease after possible incomplete initial resection was not evaluated. The study included 16 cases of persistent/recurrent PitNETs that underwent surgery. Gonadotroph tumors were the most prevalent (*n* = 6), with no detected alterations (Pattern 1) in the majority and one case showing isolated copy number gains (Pattern 3). Although the majority of lactotroph PitNETs were largely chromosomally disrupted, only one was repeatedly operated (patient 19, Pattern 4). The mammosomatotroph PitNET of patient 48 showed no chromosomal alterations (Pattern 1) in the material of first and two consequent operations. Additional somatic mutation analysis was also negative. Other reoperated tumors of the PIT1-lineage group included two sparsely granulated somatotroph tumors with Patterns 2 and 4 (patient 46 and patient 45, respectively). Five of the ten corticotroph tumors required repeated surgery. Three of these PitNETs (patients 4, 52, and 56; details in the section ‘Selected cases with advanced disease’ of the supplementary material) exhibited an outspoken aggressive behavior, and Pattern 2 alterations showed extensive chromosomal copy number losses (Fig. 3). At the same time, these 3 patients were the only ones who received radiotherapy between surgeries, of which only patient 56 received temozolomide. The other two recurrent corticotroph tumors (patients 1 and 2) showed Patterns 4 and 2 on two chromosomes, respectively.

### Time from primary to recurrent operation and follow-up time

The time from the first operation to the first reoperation averaged 60 months (median 31, range 9–208, mean SD 66.43; 95% CI 24.6–95.4). Some patients underwent operations long before the current study commenced, resulting in highly variable follow-up times, with a mean of 58 months (median 36, range 1–354, mean SD 65.94; 95% CI 41.52–75.3). Significant differences in follow-up times were observed between recurrent (mean 34 months, range 1–113) and non-recurrent (mean 127 months, range 38–316) cases (*P* < 0.0001, two-tailed Mann–Whitney U test).

For the PIT1-lineage group, the mean time from the primary to recurrent operation was 41 months (median 18, range 9–118, mean SD 51.73; 95% CI −41.57 to 123.1); for the SF1-lineage group, it was 71.5 months (median 57.5, range 16–206, mean SD 69.36; 95% CI −1.29 to 144.3); and for the TPIT-positive group, it was 70.4 months (median 40, range 10–217, mean SD 85.68; 95% CI −35.98 to 176.8). The plurihormonal tumor (patient 44) was reoperated after 16 months.

No correlation was found between the total number of alterations, the total quantity of LOH or imbalances, and the time elapsed from the primary to recurrent operations in all recurrent cases (*n* = 16).

### Comparison of primary and recurrent tumors

We compared the imbalance–LOH–CNV patterns of primary tumors with one or more recurrences in five patients (Supplementary Table 2). We found no major differences in the imbalance–LOH–CNV patterns over a time span of 2–10 years (median 2 years, mean 3.8 years), with one exception. A sparsely granulated lactotroph microtumor (patient 19) with Pattern 4 acquired an imbalance due to copy number gain on chromosome 14 during the two years elapsed between the primary and recurrent operation.

## Discussion

In our study, we utilized a validated 1500-SNP NGS panel designed for genome-wide detection of imbalances, LOH, and CNV alterations to investigate patterns of chromosomal alterations in PitNETs and place them in the context of previously reported heterogeneous data. Our aim was to validate and systematize these using an easy and affordable method, exploring the possible applications in routine diagnostics.

We confirmed the occurrence of chromosomal alterations widely described in the literature (Supplementary Fig. 2A and B) and categorized our results into specific patterns (Patterns 1–4). Pattern 3 and Pattern 4 alterations were most frequently observed in the PIT1-lineage PitNETs, while Pattern 1 alterations (no alterations) were predominantly seen in gonadotroph PitNETs. This suggests a diverse biological background in the latter, with other molecular drivers beside DNA variations or gross chromosomal changes. Kurelac *et al.* described somatic complex I disruptive mitochondrial DNA mutations as modifiers of tumorigenesis, correlating with low genomic instability in (oncocytic) PitNETs, most likely representing gonadotroph lineage in the current tumor classification (Kurelac *et al.* 2013).

Among the ten corticotroph PitNETs, nine had disrupted genomes with Pattern 2 (6×), Pattern 3 (2×), and Pattern 4 (1×). The only case with Pattern 1 was driven by a known *USP8* mutation, consistent with previously described *USP8*-mutated, genome-stable corticotroph PitNETs (Uzilov *et al.* 2021). All (*n* = 4) silent corticotroph tumors were disrupted. In addition, one metastatic corticotroph PitNET (patient 4) and two corticotroph ‘aggressive pituitary tumors’ (corticotroph APT (aggressive pituitary tumor), patients 52 and 56) were characterized by outspoken Pattern 2 alterations, exhibiting genome-wide whole chromosome copy number losses, leading to near-haploid genomes (genotype A0 or 0B) or near-homozygous genomes (NHG, genotype AA or BB), respectively. The latter occurred after endoreduplication/genome doubling and were illustrated in case 56 by WGS, which revealed an average tumor ploidy of 1.3, proving the genome doubling. The phenomenon of near-haploidization with or without endoreduplication/genome doubling and its association with aggressive disease have been described in various tumor types, including subsets of chondrosarcoma (Bovée *et al.* 2000), localized mesotheliomas (Hung *et al.* 2020), adrenal cortical cancers (Zheng *et al.* 2016), and OTCs (Corver *et al.* 2014, Ganly *et al.* 2018, Gopal *et al.* 2018).

The question arises as to whether extensive losses could be caused by aggressive treatment modalities before surgery. Among the recurrent cases in the present study, only three patients (patients 4, 52, and 56) with aggressive corticotroph tumors and extensive Pattern 2 received radiotherapy between surgeries. In addition, patient 56 received temozolomide. Previously, a cohort of corticotroph PitNETs enriched for aggressive subtypes (22 patients) was analyzed by whole-exome sequencing (Uzilov *et al.* 2021). They described genome doubling in 4/22 PitNET cases, the presence of haploid chromosomes in 6/22, and three PitNETs with a near-haploid genome. Among these PitNETs were three primary corticotroph tumors with extensive chromosomal losses (haploid), which had neither been irradiated nor subjected to chemotherapy. Uzilov and coworkers (Uzilov *et al.* 2021) reflect on the work of Bi and coworkers*.*, in which three corticotroph tumors with CNV-disrupted genomes are haploid or tetraploid (Bi *et al.* 2017). Patient 56 of our cohort with APT was treated with immune checkpoint inhibition (ICI) therapy as a last resort by longstanding aggressive disease, but unfortunately, passed away due to massive complications, including brain hemorrhage. No therapy response in tumor tissue from the autopsy material was observed, consistent with most previously reported cases of nonmetastatic aggressive corticotroph PitNETs (Ilie *et al.* 2022). Up until now, 16 corticotroph tumors were reported to be treated with ICI (Ilie *et al.* 2022). Among these are five APTs and nine metastatic PitNETs (‘carcinomas’). Of all APTs, one tumor completely responded to therapy, one showed stable disease, while another five progressed. The only APT with an excellent response to therapy was a case in which the presence of metastatic disease was not evaluated (Shah *et al.* 2022). This tumor was also DNA mismatch repair deficient (MMRd) and *TP53* mutant; however, progressive disease was described in another MMRd APT treated with ICI (Ilie *et al.* 2022). No molecular data are available of the one patient with stable disease after ICI treatment. Among metastatic corticotroph PitNETs (*n* = 9), partial response was seen in six patients, one had stable, and the other two progressed, suggesting a better therapy response in the setting of metastatic corticotroph PitNETs than in APTs.

As previously documented, the lactotroph PitNETs emerged as the most altered subgroup within the PIT1-lineage tumors (De Sousa *et al.* 2019). We further delineated distinct patterns within lactotroph subtypes, as sparsely granulated lactotrophs mostly show Pattern 4 alterations and the majority of densely granulated lactotrophs were associated with Pattern 3, with only one tumor in Pattern 1. Such subtyping might hold clinical relevance (Gomez-Hernandez *et al.* 2015). Interestingly, even being the largest subgroup, no lactotrophs were seen within Pattern 2, while both immature tumors of the PIT1 lineage and three out of four somatotroph tumors were seen within Pattern 2. Nineteen out of 20 lactotrophs exhibited chromosomal alterations, underscoring the potential utility in daily practice when histology and immunohistochemistry are inconclusive in identifying neoplasms in the resected material. In our cohort, two of such patients (60 and 61) were accurately diagnosed only after applying the imbalance–LOH–CNV assay. Similarly, chromosomal alterations were observed in the majority of the corticotroph tumors. We could not find an association of disrupted genome in lactotroph tumors with higher prolactin levels. The extent of alterations in lactotroph PitNETs did not correlate with recurrence, echoing previous observations in subtypes of OT neoplasia, where we described ‘reciprocal’ chromosomal imbalance type CNA (copy number alterations), characterized by imbalanced chromosomal copy number gains associated with benign disease, that might resemble the Pattern 3 (and Pattern 4) alterations dominantly seen in the PIT1-lineage PitNETs (de Koster *et al.* 2023). Comparison of primary and recurrent lesions in five patients showed that, with minor exceptions, alterations did not progress over time, suggesting that the genetic landscape of these tumors might remain stable despite recurrence. The latter was also seen by Uzilov and coworkers in one case (Uzilov *et al.* 2021).

In conclusion, while this study reaffirms the previously described highly intricate genomic landscape of PitNETs, the integration of genome-wide chromosomal patterns with targeted DNA variation detection in selected cases could enhance the pathological characterization of these tumors. Moreover, this approach may facilitate earlier detection of potentially aggressive tumors compared to morphological analysis, particularly in corticotroph cases, where a near-haploid/near-homozygous genome is discovered.

The clinical implications and practice points are summarized in Box 1.

Box 1 **Clinical implications and practice points for the analysis of distinct chromosomal alteration patterns in PitNETs.**

*Identification of PitNETs*: In cases of hyperprolactinemia or hypercortisolism with uncertain histology and immunohistochemistry, chromosomal pattern analysis can assist in identifying neoplastic tissue, as lactotroph and corticotroph PitNETs frequently show a disrupted genome. Lactotroph PitNETs are the only group of PitNETs for which gains have been reported on all chromosomes, and for this tumor group, chromosomal losses have also been widely documented with the exception of chromosomes 8q, 12p+q, 14, 17q, and 19p+q, which are frequently observed in corticotroph tumors. Enrichment of histological regions with suspicion is essential. The approach may help to molecularly delineate hyperplasia from PitNETs.

*Corticotroph PitNETs*: A subset of aggressive and/or metastatic corticotroph PitNETs is characterized by massive chromosomal losses (Pattern 2), leading to near-haploid or near-homozygous genomes (the latter following endoreduplication/genome doubling). With concurrent DNA variant analysis, an improved pathological and molecular classification is feasible. For corticotroph tumors with quiet genomes, consider somatic mutation analysis, as it may have therapeutic implications (e.g., *USP8*-mutated tumor tends to respond better to pasireotide).

*Gonadotroph PitNETs*: Since gonadotroph PitNETs (SF1 lineage) typically exhibit quiet genomes, chromosomal pattern analysis does not provide additional value. When alterations are observed, they are unlikely, involve massive losses, and predominantly exhibit infrequent gains.

*Resistance and treatment correlation*: Further investigation is needed to determine whether chromosomal alteration patterns can indicate resistance or sensitivity to different therapy modalities, including medication, temozolomide, or radiotherapy.

## Supplementary materials



## Declaration of interest

The authors declare that there is no conflict of interest that could be perceived as prejudicing the impartiality of this work.

## Funding

This work did not receive any specific grant from any funding agency in the public, commercial, or not-for-profit sector.

## Data availability

The authors confirm that all relevant data are available upon request.
