# Supplementary material for: Chromosomal alteration patterns in PitNETs: massive losses in aggressive tumors
Source: Endocr Relat Cancer. 2024 Dec 19;32(1):e240070. doi: 10.1530/ERC-24-0070 (PMC11729050; doi:10.1530/ERC-24-0070)
Supplement: Supplementary file 1 [file supplementary_materials.pdf]

Immunohistochemistry

Beside hematoxylin and eosin stain, the tumors were evaluated with immunohistochemical stains for standard transcription factors PIT1, TPIT, SF1 as well recently introduced by WHO classification of tumors GATA3 and ER; hormones GH, PRL, ACTH, FSH, LH, TSH, proliferation marker Ki67, cytokeratin 18, and p53 on selected cases. (Asa and Mete, 2021, Mete et al., 2018a, Mete et al., 2018b, Asa et al., 2017) The proliferation index was counted manually for at least 500 tumor cells as recommended by WHO. All labeled neoplastic cells were counted regardless of intensity. (Villa et al., 2019) Cytokeratin 18 staining was performed and evaluated as recommended by WHO

Antibody	Clone	Company	Species
ACTH	02A3	Dako	Mouse
ER (Estrogen Receptor Alpha)	EP1	Dako	Rabbit
FSH	C10	Dako	Mouse
GATA3	L50-823	Cell Marque	Mouse
Growth Hormone		Dako	Rabbit
Keratine 18	DC10	Dako	Mouse
Ki-67	MIB-1	Dako	Mouse
LH	C93	Dako	Mouse
PIT-1		Nodus	Rabbit
SF-1	EPR19744	ABCAM	Rabbit
T-PIT	CL6251	Atlas	Mouse
TSH	0042	Dako	Mouse

Supplementary Material 1, Table 1: The antibodies used in the routine diagnostics

Additional panels used for somatic mutation analysis in isolated cases

The most recent information on used panels is publicly available on: https://www.palga.nl/voor-pathologen/moleculaire-bepaling
In the drop down menu choose LUMC to see the list of panels.

NGS custom Ampliseq Cancer Hotspot v6 panel

Analysis is performed on the hotspots of ABL1; AKT1; ALK; APC; ARAF; ATM; BAP1 (exon 3-17 partieel); BRAF; CARD11; CD79A; CD79B; CDC73; CDH1; CDK4; CDKN2A; CIC; CSF1R; CTNNB1; CTNNB1; DDR; DICER; EGFR; EIF1AX; ERBB2; ERBB3; ERBB4; ERCC2; ERCC2; EZH2; FBXW7; FGFR1; FGFR2; FGFR3; FLT3; FOXL2; GNA11; GNAQ; GNAS; H3F3A; H3F3B; HNF1A; HRAS; IDH1; IDH2; JAK2; JAK3; KDR; KIT; KRAS; MAP2K1; MAP2K2; MAP2K4; MAP3K1; MDM2; MED12; MET; MLH1; MPL; MUTYH; MYC; MYD88; MyoD1; NKX2-1; NOTCH1; NTRK1; NPM1; NRAS; PDGFRA; PDGFRB; PIK3CA; POLD1; POLE; PPP2R1A; PTEN; PTK2; PTPN11; RB1; RET; SMAD4; SMARCB1; SMO; SRC; STK11; TERT-promotor; TP53 (exon 2-11 partially) and VHL. Copy Number Variation (CNV) is identified for the relevant genes and/or chromosomal regions.

NGS Custom AmpliSeq NGS BRCA v5 panel

Particial analysis is performed on the coding exons of the following genes: BRCA1, BRCA2, ATM, BARD1, BRIP1, CDK12, CHEK1, CHEK2, FANCL, PALB2, PPP2R2A, RAD51B, RAD51C, RAD51D, en RAD54L. Following parts are suboptimal covered: ATM exon 25 (c.3673-3746), exon 36 (c.5437-5462), exon 52 (c.7726-7788), CDK12 exon 1 (c.880-906), PALB2 exon 4 (c.212-258), RAD51C (c.706-723) and RAD51D (c.388-480).

Oncomine Comprehensive Assay plus (OCAplus)

Information available on: https://www.palga.nl/media/uploads/pdf/5/8/580_135-ocaplus-lumc.pdf

NGS Custom Ampliseq RCPL Panel v.1

The analysis is performed on the coding exons of the following genes:ARMC5, ATP1A1, ATP2B3, BAP1, CACNA1D, CDC73, CDH1, CDKN1A, CDKN1B, CDKN1C, CDKN2A, CDKN2B, CDKN2C, CDKN2D, CYSLTR2, DICER1, EIF1AX, KCNJ5, KIT, KRAS, LZTR1, MBD4, MEN1, MTOR, NF1,

NF2, PLCB4, PRKAR1A, PTEN, SMARCB1, SMARCE1, SRSF2, SUFU, TP53, TSC1, TSC2, U2AF1. Also following genes are analyzed: BRAF, CCAT1, CDC27, CDK4, GNA11, GNAQ, HRAS, IDH1, IDH2, NRAS, PIK3CA, PTK2, RET, SF3B1 and the TERT promotor. Loss of Heterozygosity (LOH) and Copy number variation (CNV) are evaluated for relevant genes and chromosomal regions.

References

WHO Classification of Tumours Editorial Board. Endocrine and neuroendocrine tumours. Lyon (France): International Agency for Research on Cancer; 2022. [cited 2024 02 23](WHO classification of tumours series, 5th ed.; vol. 10). https://publications.iarc.fr.

ASA, S. L., KUCHARCZYK, W. & EZZAT, S. 2017. Pituitary acromegaly: not one disease. *Endocr Relat Cancer,* 24**,** C1-c4.

ASA, S. L. & METE, O. 2021. Cytokeratin profiles in pituitary neuroendocrine tumors. *Hum Pathol,* 107**,** 87-95.

METE, O., ALSHAIKH, O. M., CINTOSUN, A., EZZAT, S. & ASA, S. L. 2018a. Synchronous Multiple Pituitary Neuroendocrine Tumors of Different Cell Lineages. *Endocr Pathol,* 29**,** 332-338.

METE, O., CINTOSUN, A., PRESSMAN, I. & ASA, S. L. 2018b. Epidemiology and biomarker profile of pituitary adenohypophysial tumors. *Mod Pathol,* 31**,** 900-909.

VILLA, C., VASILJEVIC, A., JAFFRAIN-REA, M. L., ANSORGE, O., ASIOLI, S., BARRESI, V., CHINEZU, L., GARDIMAN, M. P., LANIA, A., LAPSHINA, A. M., POLIANI, L., REINIGER, L., RIGHI, A., SAEGER, W., SOUKUP, J., THEODOROPOULOU, M., UCCELLA, S., TROUILLAS, J. & RONCAROLI, F. 2019. A standardised diagnostic approach to pituitary neuroendocrine tumours (PitNETs): a European Pituitary Pathology Group (EPPG) proposal. *Virchows Arch,* 475**,** 687-692.

Selected cases with advanced disease (patients 4, 52, and 56)

Three cases stood out due to disease progression, characterized by exceptionally persistent/recurrent disease requiring multiple operations or treatment modalities and the spread of disease locally or distant. A common feature among these patients was the presence of corticotroph tumors exhibiting near homozygous genomes.

Case 4:

This male patient, in his twenties, was diagnosed with an ACTH-producing corticotroph PitNET. 18 years elapsed between surgery on the primary tumor and the first recurrence. Subsequently, three years elapsed between the first and second recurrence, and two years between the second and third recurrence. Radiotherapy was administered for the second recurrence. Twenty-five years after the primary tumor diagnosis, a liver metastasis was discovered, followed by a fourth local recurrence two years later, which was resected. Analysis of the latter revealed a slightly elevated Ki67 proliferation index of 4,2%. Subsequent Imbalance-LOH-CNV analysis exposed Pattern 2 type of alterations (Supplementary Figure 1) with extensive imbalances due to copy number loss of whole chromosomes 1, 2, 3, 4, 5, 8, 10, 11, 15, 16, 17, 18, 21 and 22. Additionally, similar alterations were observed at the chromosomal tips of 12p and 12q. No endoreduplication/genome doubling was apparent. Targeted mutional analysis of his liver metastasis had shown a pathogenic DNA variant in *TP53*: NM_000546.5: c.586C>T, p. (Arg196*) together with LOH. Testing for DNA variants in homologous DNA recombination repair (HRD) genes (among which *BRCA1* and *BRCA2*) assessed negative. His fourth recurrence was additionally assessed and showed somatic pathogenic variations in *LZTR1*: NM_006767.4: c.373_375delGTC, p. (Val125del) with LOH and *NF1*: NM_000267.3: c.4910_4911delTT, p. (Phe1637Serfs*3).

Case 52:

At the moment of inclusion, this female patient in her sixties presented with her third recurrence of an ACTH-producing corticotroph PitNET, exhibiting a high Ki-67 proliferation index of 19,6%. The primary tumor had been diagnosed ten years earlier, with first and second recurrences occurring three and five years later, respectively. Radiotherapy was administered during the course of disease. Molecular testing was conducted on both the second and third recurrences, albeit with different assays.

Immunohistochemistry of the second recurrence suggested DNA mismatch repair proficiency, confirmed by detected microsatellite stability. Testing for Homologous Recombination Deficiency was

negative, and no targetable gene fusions were identified. However, a pathogenic splice variant in TP53 (intron 6); NM_000546.5: c.673-2A>G DNA variant was detected with LOH of the wild type allele. A pathogenic DNA variation was observed in DAXX (NM_001350.5: c.604_610delATCCGGC, p. (Ile202Glyfs*25), with LOH on the wild type allele. A monoallelic pathogenic DNA variation was found in SDHA (NM_004168.4: c.1954delGinsCCTGA, p. (Ala652Profs*3).

Furthermore, a low tumor mutation burden with 2.85 mutations per mega-base was observed. Imbalance-LOH-CNV analysis of the third recurrence (Figure 3, Panel A) with our 1500 SNP target NGS panel revealed a near homozygous genotype with multiple LOH (chromosomes 1, 2, 3, 4, part of 5q, 6, 8, 10, 11, 12, 14, 15, 16, 17, 18, 19, 20, 21, 22 and X), being consistent with possible endoreduplication. These findings align with a Pattern 2 of abnormalities, consistent with chromosomal alterations observed in the second recurrence four to five years later.

Case 56:

This male patient experienced Morbus Cushing due to a corticotroph PitNET over a span of period of 46 years. Clinically recurrent disease emerged 34 and 35 years after initial diagnosis, necessitating operative treatment. The patient had previously undergone bilateral adrenalectomy, received radiotherapy twice, and was treated with temozolomide. In 2023, a recurrence with invasion into the cavernous sinus occurred. With no further treatment options available, the medical oncologist requested whole genome sequencing (WGS) to identify potential druggable targets.

WGS of frozen tumor material from 2012 showed a DNA mismatch repair proficiency and a low mutational burden of 0.7 mutations per mega-base, suggesting no indication for immunotherapy efficacy. Proficiency in homologous DNA repair indicated no potential benefit from PARP inhibitors. No somatic cancer driver genes or pathogenic gene fusions were identified but copy number analysis showed disruption of LRP1B (on chromosome 2q) and ATRX (on chromosome Xq).

Imbalance-LOH-CNV analysis of the WGS (Figure 3, Panel B) revealed extensive chromosome loss of chromosomes 1, 2, 3, 4, 6, 8, 10, 11, 13, 15, 17, 19, 20, 21, 22, and 23, indicative of a process of “near haploidization” with subsequent endoreduplication/genome doubling, which is supported by the average tumor ploidy of 1.3.

The patient underwent experimental combined CTLA-4/PD1 blocking immunotherapy as a last resort in the absence of alternative options. Prior to therapy, the absence of metastatic disease was

confirmed radiologically. Subsequently, the patient died due to brain hemorrhage in the tumor region, in the context of anticoagulation therapy for his non-removable vena cava inferior filter. The macrotumor encased the right internal carotid and right arteria communicans. Autopsy material review revealed no signs of tumor response to the immunotherapy.

Supplementary Table 1

Type of the tumor	Total	Pattern 1	Pattern 2	Pattern 3	Pattern 4
Lactotroph	20	1	0	8	11
Mammosomatotroph	2	1	1	0	0
Somatotroph	4	0	3	0	1
Acidophil stem cell tumor	1	0	0	1	0
Immature PIT1 lineage tumor	2	0	2	0	0
Thyrotroph	1	0	0	0	1
Total	*30*	*2*	*6*	*9*	*13*

Supplementary Table 1. Distribution of patterns in the tumors of PIT1 lineage (n=30)

Supplementary Table 2

Patient<br>Sex<br>Age at Inclusion	Lineage	Histological type	GWLOH Primary: Year and Result, Pattern of Imbalance-LOH-CNV	GWLOH 1st Recurrence: Year and Result, Pattern of Imbalance-LOH-CNV	(if applicable) GWLOH Further Recurrence(s): Number, Year and Result, Pattern of Imbalance-LOH-CNV
18 (M,41)	SF1	Gonadotroph	2018 – no chromosomal alterations	2020 – no chromosomal alterations	-
19 (F,24)	PIT1	Sparsely granulated lactotroph	2019 – copy neutral LOH on chromosome 2. Imbalances due to copy number	2021 – copy neutral LOH on chromosome 2. Imbalances due to copy number	-

			gain on chromosomes 3, 5, 7, 8, 9, 11, 12, 17, 19 and 20	gain on chromosomes 3, 5, 7, 9, 11, 12, **14,** 17, 19 and 20	
45 (F,22)	PIT1	Sparsely granulated somatotroph	2020 – Imbalance due to copy number gain on chromosome 4q, Imbalance due to copy number loss on chromosome 8	2022 – Imbalance due to copy number gain on chromosome 4q, Imbalance due to copy number loss on chromosome 8	-
48 (M,49)	PIT1	Mammosomatotroph	2019 – no chromosomal alterations	2020 – no chromosomal alterations	2021– 2nd recurrence, no chromosomal alterations. Negative somatic mutation analysis (Oncomine panel, for details see Supplementary Material 1)
52 (F,60)	TPIT	Sparsely granulated corticotroph	2013: LOH on chromosomes 1, 2, 3, 4, 6, 8, 10, 11, 12, 14, 15, 16, 17, 18,	2016 – no molecular testing performed	2018 – 3rd recurrence: LOH on chromosomes 1, 2, 3, 4, 5q, 6, 8, 10, 11, 12, 14, 15,

			19, 20, 21, 22 with genotypes AA/BB after possible endoreduplication. Chromosome 5 with difficult interpretable alterations. Imbalance on chromosome X with genotype AAB.		16, 17, 18, 19, 20, 21, 22 and X. Near haploid genome with possible consequent endoreduplication 2023 – 4th recurrence: LOH on chromosomes 1, 2, 3, 4, 5q, 6, 8, 10, 11, 12, 14, 15, 16, 17, 18, 19, 20, 21, 22 and X. Identical alterations with previous recurrence of 2018.

Supplementary Table 2. Test results of recurrent cases. Comparison of the Imbalance-LOH-CNV Patterns of primary tumors with one or more operated recurrences in five patients.

Supplementary Figure 1

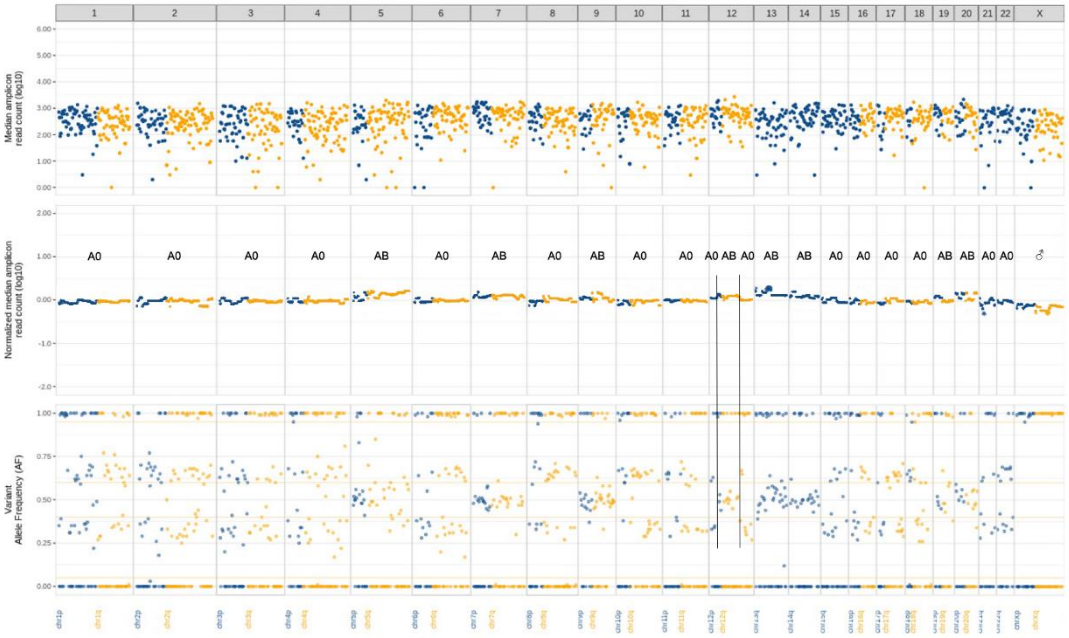

Supplementary Figure 1: GW-LOH testing and Imbalance-LOH-CNV analysis on tumor of patient 4. GW-LOH testing and subsequent Imbalance-LOH-CNV analysis showed a Pattern 2 type of abnormalities. With an estimated tumor percentage of 60% the amplitude of the imbalance/LOH pattern is lower than what seen in higher tumor percentages. No genome doubling/endoreduplication was concluded. Targeted mutional analysis of his liver metastasis had shown a pathogenic DNA variant in TP53: NM_000546.5:c.586C>T, p. (Arg196*) together with LOH. Testing for DNA variants in homologous DNA recombination repair (HRD) genes (among which BRCA1 and BRCA2) tested negative. His fourth recurrence was additionally tested and showed somatic pathogenic variations in LZTR1: NM_006767.4: c.373_375delGTC, p.(Val125del) with LOH and NF1: NM_000267.3: c.4910_4911delTT, p.(Phe1637Serfs*3). In this local recurrence surprisingly the TP53: c.586C>T DNA variation was not found, possibly pointing at clonal divergence.

Supplementary Figure 2a

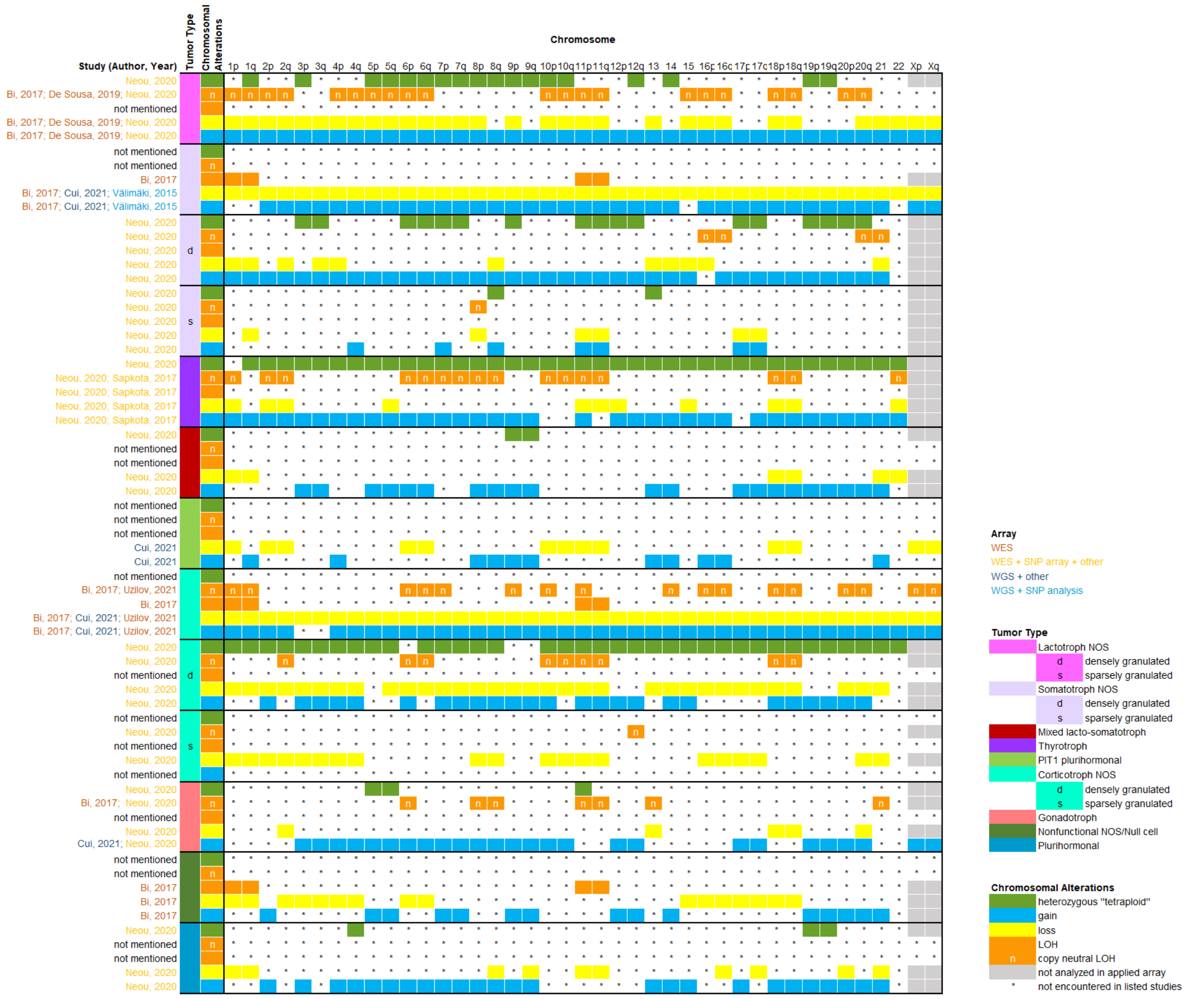

Supplementary Figure 2a. Cumulative qualitative description of previously reported Imbalances-LOH-CNV in PitNETs. The figure is based on available and clearly to extrapolate data per chromosome and tumor type of selected studies using WGS, WES and SNP based arrays (Cui et al., 2021, Bi et al., 2017b, Uzilov et al., 2021, Neou et al., 2020, Välimäki et al., 2015, Sapkota et al., 2017, De Sousa et al., 2019). Multiple authors reported varying proportions of 'genomically silent' tumors without any chromosomal alterations/CNV within every type of PitNETs, which are not depictured in the figure. The current Figure provides no reported numbers of patients with a specific type of the tumor or actual numbers of alterations; the table serves solely to depict whether certain chromosomal alteration was previously reported on certain chromosomes.

Multiple studies using conventional cytogenetic methods as comparative genomic hybridization (CGH), fluorescence in situ hybridization (FISH), or microsatellite analysis were excluded primarily due to methodological limitations in detecting copy neutral LOH (Dong et al., 1997, Bates et al., 1997,

Farrell et al., 1997, Huizenga et al., 1998, Buch et al., 2004, Clayton et al., 2000, Simpson et al., 2003, Tanaka et al., 1998, Asteria et al., 2001, Filopanti et al., 2004, Wierinckx et al., 2007, Wierinckx et al., 2018, Wierinckx et al., 2011, Cai et al., 2013, Lasolle et al., 2020, Kurelac et al., 2013). Furthermore, there was a number of relevant studies in which coupling observed alterations to tumor types or possible genotypes was difficult even after studying supplemental material (Song et al., 2016, Tatsi et al., 2019, Chen et al., 2020, Zhang et al., 2022).

Notice the transition of tumor classification with the introduced application of transition factors. Obviously, all types of alterations were previously described in most common tumor types. In earlier cohorts, nonfunctional/null cell group contained often multiple tumors with an undisrupted genome or few copy number gains consistent with modern clustering of SF1 lineage PitNETs, and tumors with extensive alterations suggesting the prevalence in this group of silent corticotroph tumors or nonfunctional tumors of other lineages before the wide diagnostic application of transcription factors. Remarkably, no genome-wide molecular studies were found using complete morphological subtyping for all different types of PitNETs as recommended by WHO, which would reflect clinical observations of adversity in some of them. The most extensive morphological tumor subtyping was conducted by Neou et al., including subtypes of corticotroph and somatotroph tumors (Neou et al., 2020). Interestingly, the authors describe lactotroph tumors as rare, consisting also of 16 out of 134 tumors in the cohort. No molecular studies carefully subtyping lactotroph tumors were found to date.

Even though some studies were not included in the current figure, they provided interesting insights. In invasive tumors, higher frequencies of LOH were reported on chromosomes 11, 10 and 13 (Bates et al., 1997). Chromosome 1 showed an LOH frequency above 30% in non-functioning tumors independent of invasion status (Simpson et al., 2003). In general, PitNETs tend to harbor chromosomal gains more frequently than losses with most frequent chromosomal gains involving chromosomes 7p and 19p, followed by 19q, 7q, and 8q (Peculis et al., 2021). In cases with extensive gains, chromosomal ‘tetraploidy’ targeting several chromosomal arms to entire chromosomes are frequently reported. The most common chromosome losses in PitNETs were reported on chromosomes 1p, 11 and 22q (Bi et al., 2017a, Pack et al., 2005, Bi et al., 2017b) followed by 1q, 2, and 18. (Bi et al., 2017b). Less frequent lost chromosomes are 4, 7, 10, 14, and 15. (Bi et al., 2017b). Tumors exhibiting whole genome doubling are reported to show loss on chromosome 1 (Bi et al.,

2017b). Other frequent regions of loss involve chromosome 17, 16p , 4, 10p, 12, 20, 13q, and 9p. (Pack et al., 2005)

A significant association between disrupted genome in PitNETs and functional hormone status was reported, but no association with atypical histopathology, proliferative index, or recurrence was found (Bi et al., 2017a, Neou et al., 2020). Higher overall LOH frequency was reported to be associated with both tumor recurrence and invasiveness compared with noninvasive counterparts (Buch et al., 2004, Simpson et al., 2003).

Clinically silent tumors are more likely to be gonadotroph tumors with infrequent chromosomal copy number variations (CNV) (Neou et al., 2020, Bi et al., 2017b). Silent somatotroph and corticotroph tumors with quiet genome were reported (Neou et al., 2020). Functioning somatotroph tumors more often show CNV with chromosomal losses being associated with invasive tumors. For corticotroph tumors near haploid genomes with whole chromosome losses of many chromosomes and genome doubling, but also cases with widespread copy number gains of many chromosomes were described (Uzilov et al., 2021, Bi et al., 2017b). High levels of aneuploidy were associated with macrotumor and invasiveness independent of *USP8* and *TP53* status (Uzilov et al., 2021). Rare thyrotroph tumors showed CNV in 62.5% in a small cohort (n=12)(Sapkota et al., 2017). Lactotroph tumors were reported as highly disrupted with more chromosomal gains than losses with variable associated aggressiveness (Neou et al., 2020, De Sousa et al., 2019). A highly disrupted PitNET genome may be associated with recurrence, but no specific CNV was found as a prognostic marker (Lasolle et al., 2020). Loss of chromosome 11 was reported in two aggressive and three metastatic lactotroph PitNETs (Wierinckx et al., 2011)**.** Extensive copy number gains are possibly associated with higher prolactin production and bromocriptine resistance (Chen et al., 2020). A majority of PitNETs exhibit a predominant form of copy number alterations, with either gains or losses, and with a minority of tumors showing both or none. These changes seem to play a crucial role in the biology of PitNETs with mechanisms yet to be identified.

Supplementary Figure 2b

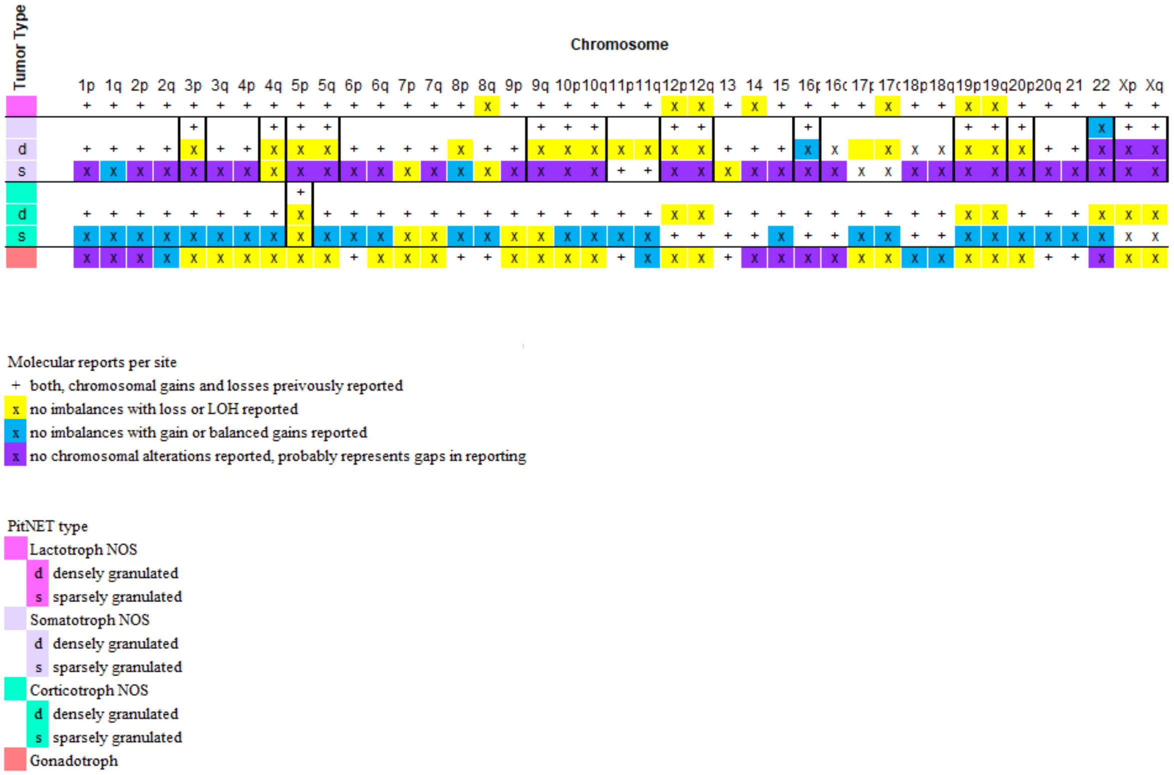

Supplementary Figure 2b. Summarized version of Figure 2a, integrating findings with previous studies, focused on previously unreported alterations. Mixed tumors and null-cell/nonfunctional NOS were excluded due to uncertainty in tumor subclassification or lineage. Some studies did not further subclassify corticotroph and somatotroph tumors, leading to some problematic areas which are highlighted with thick lines. An alteration is noted on NOS lines only when it was not previously observed in a specific subtype but was reported in “NOS” subtype.

Lactotroph tumors: As previously mentioned, we found no molecular studies which would specifying between sparsely or densely granulated lactotroph tumors, so the findings integrated as NOS. For alterations in subtypes see Figure 1. This is the only group of PitNETs for which gains have been reported on all chromosomes. Chromosomal losses have also been widely reported, with the exception of chr. 8q, 12p+q, 14, 17q, and 19p+q.

Somatotroph tumors: These tumors have rarely been subclassified, but some patterns can be observed. Densely granulated tumors predominantly show gains or losses, but no losses have been reported on reported on chromosomes 8p, 11p+q, and 17p+q. Sparsely granulated somatotrophs are

more likely to gain 8p, and exhibit losses on 11p+q and 17p+q. Chromosomal alterations in sparsely granulated somatotroph tumors are likely widely underreported.

Corticotroph tumors: These tend to show almost a clear relationship between histological subtype and molecular profile. Sparsely granulated corticotrophs frequently show extensive losses and not reported to gain 1pq, 2pq, 3pq, 4pq, 5q, 6pq, 8pq, 10pq, 11pq, 15, 17pq, 19pq, 20pq, 21, and 22. Densely granulated tumors, however, tend to show less chromosomal alterations and reported to show gains or losses on these chromosomes, except LOH 19p+q and 22, which are not reported for densely granulated tumors.

Gonadotroph tumors: These tumors usually exhibit a largely silent genome, but when alterations are observed, they tend to show chromosomal gains. Although gains have been widely reported, losses have not been documented on multiple chromosomes.

References

WHO Classification of Tumours Editorial Board. Endocrine and neuroendocrine tumours. Lyon (France): International Agency for Research on Cancer; 2022. [cited 2024 02 23](WHO classification of tumours series, 5th ed.; vol. 10). https://publications.iarc.fr.

ASTERIA, C., ANAGNI, M., PERSANI, L. & BECK-PECCOZ, P. 2001. Loss of heterozygosity of the MEN1 gene in a large series of TSH-secreting pituitary adenomas. *J Endocrinol Invest,* 24**,** 796-801.

BATES, A. S., FARRELL, W. E., BICKNELL, E. J., MCNICOL, A. M., TALBOT, A. J., BROOME, J. C., PERRETT, C. W., THAKKER, R. V. & CLAYTON, R. N. 1997. Allelic deletion in pituitary adenomas reflects aggressive biological activity and has potential value as a prognostic marker. *J Clin Endocrinol Metab,* 82**,** 818-24.

BI, W. L., GREENWALD, N. F., RAMKISSOON, S. H., ABEDALTHAGAFI, M., COY, S. M., LIGON, K. L., MEI, Y., MACCONAILL, L., DUCAR, M., MIN, L., SANTAGATA, S., KAISER, U. B., BEROUKHIM, R., LAWS, E. R., JR. & DUNN, I. F. 2017a. Clinical Identification of Oncogenic Drivers and Copy-Number Alterations in Pituitary Tumors. *Endocrinology,* 158**,** 2284-2291.

BI, W. L., HOROWITZ, P., GREENWALD, N. F., ABEDALTHAGAFI, M., AGARWALLA, P. K., GIBSON, W. J., MEI, Y., SCHUMACHER, S. E., BEN-DAVID, U., CHEVALIER, A., CARTER, S., TIAO, G.,

BRASTIANOS, P. K., LIGON, A. H., DUCAR, M., MACCONAILL, L., LAWS, E. R., JR., SANTAGATA, S., BEROUKHIM, R. & DUNN, I. F. 2017b. Landscape of Genomic Alterations in Pituitary Adenomas. *Clin Cancer Res,* 23**,** 1841-1851.

BUCH, H. N., RASKAUSKIENE, D., BAHAR, A., BICKNELL, E. J., FARRELL, W. E. & CLAYTON, R. N. 2004. Prediction of recurrence of nonfunctioning pituitary tumours by loss of heterozygosity analysis. *Clin Endocrinol (Oxf),* 61**,** 19-25.

CAI, F., ZHANG, Y. D., ZHAO, X., YANG, Y. K., MA, S. H., DAI, C. X., LIU, X. H., YAO, Y., FENG, M., WEI, J. J., XING, B., JIAO, Y. H., WEI, Z. Q., YIN, Z. M., ZHANG, B., GU, F. & WANG, R. Z. 2013. Screening for AIP gene mutations in a Han Chinese pituitary adenoma cohort followed by LOH analysis. *Eur J Endocrinol,* 169**,** 867-84.

CHEN, Y., GAO, H., XIE, W., GUO, J., FANG, Q., ZHAO, P., LIU, C., ZHU, H., WANG, Z., WANG, J., GUI, S., ZHANG, Y. & LI, C. 2020. Genomic and transcriptomic analysis of pituitary adenomas reveals the impacts of copy number variations on gene expression and clinical prognosis among prolactin-secreting subtype. *Aging (Albany NY),* 13**,** 1276-1293.

CLAYTON, R. N., PFEIFER, M., ATKINSON, A. B., BELCHETZ, P., WASS, J. A., KYRODIMOU, E., VANDERPUMP, M., SIMPSON, D., BICKNELL, J. & FARRELL, W. E. 2000. Different patterns of allelic loss (loss of heterozygosity) in recurrent human pituitary tumors provide evidence for multiclonal origins. *Clin Cancer Res,* 6**,** 3973-82.

CUI, Y., LI, C., JIANG, Z., ZHANG, S., LI, Q., LIU, X., ZHOU, Y., LI, R., WEI, L., LI, L., ZHANG, Q., WEN, L., TANG, F. & ZHOU, D. 2021. Single-cell transcriptome and genome analyses of pituitary neuroendocrine tumors. *Neuro Oncol,* 23**,** 1859-1871.

DE SOUSA, S. M. C., WANG, P. P. S., SANTORENEOS, S., SHEN, A., YATES, C. J., BABIC, M., ESHRAGHI, L., FENG, J., KOSZYCA, B., ROBERTS-THOMSON, S., SCHREIBER, A. W., TORPY, D. J. & SCOTT, H. S. 2019. The Genomic Landscape of Sporadic Prolactinomas. *Endocr Pathol,* 30**,** 318-328.

DONG, Q., DEBELENKO, L. V., CHANDRASEKHARAPPA, S. C., EMMERT-BUCK, M. R., ZHUANG, Z., GURU, S. C., MANICKAM, P., SKARULIS, M., LUBENSKY, I. A., LIOTTA, L. A., COLLINS, F. S.,

MARX, S. J. & SPIEGEL, A. M. 1997. Loss of heterozygosity at 11q13: analysis of pituitary tumors, lung carcinoids, lipomas, and other uncommon tumors in subjects with familial multiple endocrine neoplasia type 1. *J Clin Endocrinol Metab,* 82**,** 1416-20.

FARRELL, W. E., SIMPSON, D. J., BICKNELL, J. E., TALBOT, A. J., BATES, A. S. & CLAYTON, R. N. 1997. Chromosome 9p deletions in invasive and noninvasive nonfunctional pituitary adenomas: the deleted region involves markers outside of the MTS1 and MTS2 genes. *Cancer Res,* 57**,** 2703-9.

FILOPANTI, M., BALLARÈ, E., LANIA, A. G., BONDIONI, S., VERGA, U., LOCATELLI, M., ZAVANONE, L. M., LOSA, M., GELMINI, S., PERI, A., ORLANDO, C., BECK-PECCOZ, P. & SPADA, A. 2004. Loss of heterozygosity at the SS receptor type 5 locus in human GH- and TSH-secreting pituitary adenomas. *J Endocrinol Invest,* 27**,** 937-42.

HUIZENGA, N. A., DE LANGE, P., KOPER, J. W., CLAYTON, R. N., FARRELL, W. E., VAN DER LELY, A. J., BRINKMANN, A. O., DE JONG, F. H. & LAMBERTS, S. W. 1998. Human adrenocorticotropin-secreting pituitary adenomas show frequent loss of heterozygosity at the glucocorticoid receptor gene locus. *J Clin Endocrinol Metab,* 83**,** 917-21.

KURELAC, I., MACKAY, A., LAMBROS, M. B., DI CESARE, E., CENACCHI, G., CECCARELLI, C., MORRA, I., MELCARNE, A., MORANDI, L., CALABRESE, F. M., ATTIMONELLI, M., TALLINI, G., REIS-FILHO, J. S. & GASPARRE, G. 2013. Somatic complex I disruptive mitochondrial DNA mutations are modifiers of tumorigenesis that correlate with low genomic instability in pituitary adenomas. *Hum Mol Genet,* 22**,** 226-38.

LASOLLE, H., ELSENSOHN, M. H., WIERINCKX, A., ALIX, E., BONNEFILLE, C., VASILJEVIC, A., CORTET, C., DECOUDIER, B., STURM, N., GAILLARD, S., FERRIÈRE, A., ROY, P., JOUANNEAU, E., BERTOLINO, P., BARDEL, C., SANLAVILLE, D. & RAVEROT, G. 2020. Chromosomal instability in the prediction of pituitary neuroendocrine tumors prognosis. *Acta Neuropathol Commun,* 8**,** 190.

NEOU, M., VILLA, C., ARMIGNACCO, R., JOUINOT, A., RAFFIN-SANSON, M. L., SEPTIER, A., LETOURNEUR, F., DIRY, S., DIEDISHEIM, M., IZAC, B., GASPAR, C., PERLEMOINE, K., VERJUS, V., BERNIER, M., BOULIN, A., EMILE, J. F., BERTAGNA, X., JAFFREZIC, F., LALOE, D., BAUSSART, B., BERTHERAT, J., GAILLARD, S. & ASSIÉ, G. 2020. Pangenomic Classification of Pituitary Neuroendocrine Tumors. *Cancer Cell,* 37**,** 123-134.e5.

PACK, S. D., QIN, L. X., PAK, E., WANG, Y., AULT, D. O., MANNAN, P., JAIKUMAR, S., STRATAKIS, C. A., OLDFIELD, E. H., ZHUANG, Z. & WEIL, R. J. 2005. Common genetic changes in hereditary and sporadic pituitary adenomas detected by comparative genomic hybridization. *Genes Chromosomes Cancer,* 43**,** 72-82.

PECULIS, R., NIEDRA, H. & ROVITE, V. 2021. Large Scale Molecular Studies of Pituitary Neuroendocrine Tumors: Novel Markers, Mechanisms and Translational Perspectives. *Cancers (Basel),* 13.

SAPKOTA, S., HORIGUCHI, K., TOSAKA, M., YAMADA, S. & YAMADA, M. 2017. Whole-Exome Sequencing Study of Thyrotropin-Secreting Pituitary Adenomas. *J Clin Endocrinol Metab,* 102**,** 566-575.

SIMPSON, D. J., BICKNELL, E. J., BUCH, H. N., CUTTY, S. J., CLAYTON, R. N. & FARRELL, W. E. 2003. Genome-wide amplification and allelotyping of sporadic pituitary adenomas identify novel regions of genetic loss. *Genes Chromosomes Cancer,* 37**,** 225-36.

SONG, Z. J., REITMAN, Z. J., MA, Z. Y., CHEN, J. H., ZHANG, Q. L., SHOU, X. F., HUANG, C. X., WANG, Y. F., LI, S. Q., MAO, Y., ZHOU, L. F., LIAN, B. F., YAN, H., SHI, Y. Y. & ZHAO, Y. 2016. The genome-wide mutational landscape of pituitary adenomas. *Cell Res,* 26**,** 1255-1259.

TANAKA, C., KIMURA, T., YANG, P., MORITANI, M., YAMAOKA, T., YAMADA, S., SANO, T., YOSHIMOTO, K. & ITAKURA, M. 1998. Analysis of loss of heterozygosity on chromosome 11 and infrequent inactivation of the MEN1 gene in sporadic pituitary adenomas. *J Clin Endocrinol Metab,* 83**,** 2631-4.

TATSI, C., PANKRATZ, N., LANE, J., FAUCZ, F. R., HERNÁNDEZ-RAMÍREZ, L. C., KEIL, M., TRIVELLIN, G., CHITTIBOINA, P., MILLS, J. L., STRATAKIS, C. A. & LODISH, M. B. 2019. Large Genomic Aberrations in Corticotropinomas Are Associated With Greater Aggressiveness. *J Clin Endocrinol Metab,* 104**,** 1792-1801.

UZILOV, A. V., TAIK, P., CHEESMAN, K. C., JAVANMARD, P., YING, K., ROEHNELT, A., WANG, H., FINK, M. Y., LAU, C. Y., MOE, A. S., VILLAR, J., BEDERSON, J. B., STEWART, A. F., DONOVAN, M. J., MAHAJAN, M., SEBRA, R., POST, K. D., CHEN, R. & GEER, E. B. 2021. USP8 and TP53 Drivers are Associated with CNV in a Corticotroph Adenoma Cohort Enriched for Aggressive Tumors. *J Clin Endocrinol Metab,* 106**,** 826-842.

VÄLIMÄKI, N., DEMIR, H., PITKÄNEN, E., KAASINEN, E., KARPPINEN, A., KIVIPELTO, L., SCHALIN-JÄNTTI, C., AALTONEN, L. A. & KARHU, A. 2015. Whole-Genome Sequencing of Growth Hormone (GH)-Secreting Pituitary Adenomas. *J Clin Endocrinol Metab,* 100**,** 3918-27.

WIERINCKX, A., AUGER, C., DEVAUCHELLE, P., REYNAUD, A., CHEVALLIER, P., JAN, M., PERRIN, G., FÈVRE-MONTANGE, M., REY, C., FIGARELLA-BRANGER, D., RAVEROT, G., BELIN, M. F., LACHUER, J. & TROUILLAS, J. 2007. A diagnostic marker set for invasion, proliferation, and aggressiveness of prolactin pituitary tumors. *Endocr Relat Cancer,* 14**,** 887-900.

WIERINCKX, A., DELGRANGE, E., BERTOLINO, P., FRANÇOIS, P., CHANSON, P., JOUANNEAU, E., LACHUER, J., TROUILLAS, J. & RAVEROT, G. 2018. Sex-Related Differences in Lactotroph Tumor Aggressiveness Are Associated With a Specific Gene-Expression Signature and Genome Instability. *Front Endocrinol (Lausanne),* 9**,** 706.

WIERINCKX, A., ROCHE, M., RAVEROT, G., LEGRAS-LACHUER, C., CROZE, S., NAZARET, N., REY, C., AUGER, C., JOUANNEAU, E., CHANSON, P., TROUILLAS, J. & LACHUER, J. 2011. Integrated genomic profiling identifies loss of chromosome 11p impacting transcriptomic activity in aggressive pituitary PRL tumors. *Brain Pathol,* 21**,** 533-43.

ZHANG, F., ZHANG, Q., ZHU, J., YAO, B., MA, C., QIAO, N., HE, S., YE, Z., WANG, Y., HAN, R., FENG, J., WANG, Y., QIN, Z., MA, Z., LI, K., ZHANG, Y., TIAN, S., CHEN, Z., TAN, S., WU, Y., RAN, P.,

WANG, Y., DING, C. & ZHAO, Y. 2022. Integrated proteogenomic characterization across major histological types of pituitary neuroendocrine tumors. *Cell Res,* 32**,** 1047-1067.
